# AI Prediction of Neuropathic Pain after Lumbar Disc Herniation—Machine Learning Reveals Influencing Factors

**DOI:** 10.3390/biomedicines10061319

**Published:** 2022-06-04

**Authors:** André Wirries, Florian Geiger, Ahmed Hammad, Martin Bäumlein, Julia Nadine Schmeller, Ingmar Blümcke, Samir Jabari

**Affiliations:** 1 Spine Center, Hessing Foundation, Hessingstrasse 17, 86199 Augsburg, Germany; florian.geiger@hessing-stiftung.de (F.G.); ahmed.hammad@hessing-stiftung.de (A.H.); 2 Center for Orthopaedics and Trauma Surgery, Philipps University of Marburg, Baldingerstrasse, 35043 Marburg, Germany; baeumlei@med.uni-marburg.de; 3 Neuropathological Institute, University Hospitals Erlangen, Schwabachanlage 6, 91054 Erlangen, Germany; julia.nadine.schmeller@fau.de (J.N.S.); ingmar.bluemcke@uk-erlangen.de (I.B.); samir.jabari@uk-erlangen.de (S.J.)

**Keywords:** artificial intelligence, machine learning, supervised learning, neuropathic pain, lumbar disc herniation, conservative, operative

## Abstract

The treatment options for neuropathic pain caused by lumbar disc herniation have been debated controversially in the literature. Whether surgical or conservative therapy makes more sense in individual cases can hardly be answered. We have investigated whether a machine learning-based prediction of outcome, regarding neuropathic pain development, after lumbar disc herniation treatment is possible. The extensive datasets of 123 consecutive patients were used to predict the development of neuropathic pain, measured by a visual analogue scale (VAS) for leg pain and the Oswestry Disability Index (ODI), at 6 weeks, 6 months and 1 year after treatment of lumbar disc herniation in a machine learning approach. Using a decision tree regressor algorithm, a prediction quality within the limits of the minimum clinically important difference for the VAS and ODI value could be achieved. An analysis of the influencing factors of the algorithm reveals the important role of psychological factors as well as body weight and age with pre-existing conditions for an accurate prediction of neuropathic pain. The machine learning algorithm developed here can enable an assessment of the course of treatment after lumbar disc herniation. The early, comparative individual prediction of a therapy outcome is important to avoid unnecessary surgical therapies as well as insufficient conservative therapies and prevent the chronification of neuropathic pain.

## 1. Introduction

Lumbar disc herniations may cause neuropathic pain due to persistent compressive effects on neural structures. The risk of developing chronic neuropathic pain is closely related to the duration and intensity of the symptoms. To reduce these complaints, treatment by means of conservative and surgical therapy is still controversially discussed in the current literature [1,2,3,4,5]. While a decision for surgical therapy seems reasonable in the case of severe neurological deficits due to paresis and/or sensory deficits, radicular pain can often be treated well with conservative means, and the threshold of the individual patient’s burden that justifies surgical therapy is difficult to define.

In recent years, numerous studies have reported that the results of both treatment options converge within about one year after the onset of symptoms [1,2,6]. Looking at the details, it should be noted that in most of these studies, a design has to be followed that includes patients only after a certain period of persistent symptoms, usually at least 6 weeks, sometimes up to 12 weeks. Patients who were treated with definitive therapy, e.g., surgery, before this time point, are not eligible for study inclusion and thus are not represented in these clinical trials. Therefore, the majority of existing studies describe a patient population that can tolerate at least 6 weeks of conservative therapy. It is also rarely defined how conservative therapy is performed in detail, and most studies do not define a maximum symptom duration at study inclusion. As a result, patients with acute as well as patients with already chronic symptoms are mixed in the study populations. The result is often a low discriminatory power in the results [7].

Based on these scientific findings, conservative therapy is usually recommended initially for all lumbar disc herniations, and only if this remains unsuccessful, after weeks to months, will a surgical therapy be considered [4]. However, in daily practice, there is a significant proportion of patients who are not able to tolerate such a long period of conservative therapy. It is precisely these patients who are at high risk of developing chronic neuropathic pain if these severe complaints cannot be significantly improved within an adequate time frame. Even surgical therapy can hardly prevent this chronification process if the timing chosen is too late. In these cases, there is an increasing evidence that early surgical therapy can significantly minimize long-term conditions [4,8].

However, it is difficult to differentiate which patients can benefit from early surgical therapy and in which cases surgery should be avoided as it will not provide any additional benefit. The capabilities of artificial intelligence algorithms available today can provide assistance with these types of questions. We therefore investigated, with a comprehensive prospective dataset, whether the application of machine learning can predict the course of neuropathic pain development after lumbar disc herniation treated with surgical or conservative therapy and analyzed the most relevant influencing factors.

## 2. Materials and Methods

The following methods and materials are based on the established methods in the associated pilot study, as we have published previously [9].

### 2.1. Study Design and Patient Population

Patient data were collected as part of an ongoing prospective observational study at the Hessing Spine Center in Augsburg, Germany. As part of a preliminary controlled randomized study in 2019 and 2020, almost 100% of the patients included refused randomization, therefore the study design was adapted to a prospective observational study. Accordingly, the guidelines for “Strengthening the Reporting of Observational Studies in Epidemiology” (STROBE) were applied to the study reported here.

Overall, 146 consecutive patients that were treated between May 2020 and May 2021 met the inclusion criteria, of which 6 patients declined to participate. Therefore, complete datasets of 140 patients were included. Pseudonymization of the patient data was ensured for further allocation within the framework of the follow-up examinations. From these 140 patients, the data of 17 patients were excluded who either withdrew their consent to study participation or publication of data during the course of the study (4 patients) or did not show up for follow-up (13 patients). Thus, 123 complete patient records were available for further processing. All patients provided informed written consent to the use of their data in the study and to the publication of the results. The demographic data of these patients are detailed in Table 1 and Table 2. 

Digital processing and analysis of the datasets was performed by the digital pathology and AI working group of the Department of Neuropathology at the University of Erlangen, Germany.

An approval of the local ethics committee has been obtained in advance (Ethics Committee No. 16098 of the Bavarian Medical Association, Munich, Germany). The study was registered in the German Clinical Trials Register prior to inclusion of the first patient (DRKS-ID DRKS00017595).

### 2.2. Study Inclusion Criteria

All patients included were at least 18 years old and suffered from radicular pain caused by a herniated disc of the lumbar spine, confirmed by a matching finding in an MRI scan. At the time of study inclusion, the radicular symptoms did not last longer than 12 weeks. No minimum time for the duration of the symptoms was defined so patients could be enrolled from the day the symptoms started. Exclusion criteria were instability or scoliosis in the segment of the herniated disc, advanced degeneration, a recurrent disc herniation and previously performed surgery on the affected or adjacent segments.

### 2.3. Therapy Sequence

Every patient was treated according to a standardized protocol. Conservative treatment was carried out over a period of 3–5 days in an inpatient setting, with individually adaptable therapy modules. In all cases, therapy consisted of analgesic medication, based on the WHO regimen including opioids if necessary, daily specific spinal infiltrations (all conservatively treated patients received at least one epidural or periradicular infiltration and, if necessary, supplementary facet infiltrations) and daily physiotherapy-guided exercises, balneophysical applications, followed by outpatient therapy after discharge. Surgical treatment was standardly performed as microscopically or endoscopically assisted interlaminar or translaminar sequestrectomy. Patients undergoing surgery were instructed to take physical rest for 4 weeks, after which physiotherapeutically assisted exercises could take place if the individual patient preferred.

### 2.4. Content and Structure of Learning and Test Group

Basic demographic data, as well as the MOS 36-Item Short Form Survey (SF-36) [10], the Oswestry Disability Index (ODI) [11] along with leg and back pain, each measured on a 100 mm visual analogue scale (VAS), and the Hospital Anxiety and Depression Scale (HADS) [12] for every patient were assessed on the day of admission. The target variable was defined as a set of variables consisting of the ODI score and the VAS value at 6 weeks, 6 months and 1 year after intervention. Special attention was paid from the beginning to the completeness of the data, as we believe that the success of a machine learning training strongly depends on the quality of the data sets collected. Any insufficient data were completed promptly together with the patients. If a patient would refuse follow-up, the entire data set would be excluded from the study. This was the case in 13 patients.

### 2.5. Artificial Intelligence-Based Prediction Model

The data collected from the 123 patients were stored in a comma separated value formatted file (csv). This file was read by the pandas python package (pandas v.0.23.1; python 3.6.7) [13]. Plotting of correlation matrix (matplotlib v.2.1.2 and seaborn v.0.8.1) [14], density distributions and histograms of various parameters as well as basic statistical operations were performed on the dataset. For further machine learning processing, we defined the ODI score 6, 12 and 24 months as well as leg pain 6, 12 and 24 months after the start of treatment as the target values for prediction, so the machine learning problem was a linear regression problem. By applying recursive feature elimination, weighing of feature importance and analysis of intercorrelating features, some of the parameters within the csv file for a given patient were dropped in order to reduce complexity resulting in the final features fed to the model [15]. The parameters finally used to train the neural network after recursive feature elimination was applied, are shown in Table 3. After identification of categorical variables and continuous variables, categorical variables were encoded using fastai’s tabular class. Various machine learning algorithms were cross-checked regarding their performance in tenfold cross-validation (Table 4). A simple decision tree regressor was identified to be most promising. This approach was further targeted and evaluated as follows. Further grid searching of various parameters was performed and all training was evaluated by tenfold cross-validation (Table 5 and Table 6). Finally, after training was complete, we evaluated the model predictions of the target values at the given timepoints.

## 3. Results

### 3.1. Prediction Accuracy

After the decision tree regressor algorithm produced the best values for the combined overall prediction of the different target variables in the comparison of different machine learning algorithms (see Table 4 and Table 5), the prediction values for each target variable were analyzed. Table 6 gives a detailed overview of the exact values of the mean absolute errors in the context of the 10-fold cross-validation for each of the three predictions for the VAS and the ODI values. In the cross-validation, mean absolute errors of 1.79 to 1.97 were obtained for the VAS predictions. The ODI predictions had a mean absolute error of 8.68 to 10.02. With very low values of the standard deviations of 0.31 to 0.40 for the VAS values and 1.29 to 1.75 for the ODI values, the fluctuations around the respective mean error are low and the prediction values thus achieve a stable quality level (Table 6).

Thus, the algorithm used here can enable an everyday clinical statement about the development of neuropathic leg pain after a lumbar disc herniation.

### 3.2. Feature Importance

**Figure 1:**
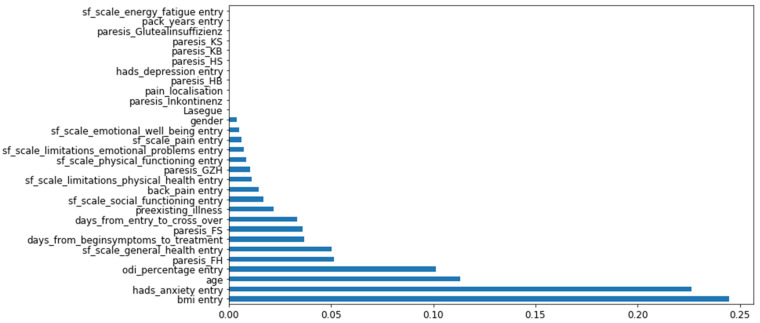
Feature importance shown according to the influence on the prediction values. Greatest influence is depicted with the greatest horizontal bar.

An analysis of the algorithm used here permits a ranking for the parameters used according to their influence on the prediction values. This ranking of the parameters is shown as an overview in Figure 1. Here, the most important parameter is the patient’s BMI at the beginning of therapy, followed by the HADS anxiety score and age. The extent of the influence on the prediction of these parameters is indicated by the horizontal bars.

### 3.3. Decision Tree Structure

**Figure 2:**
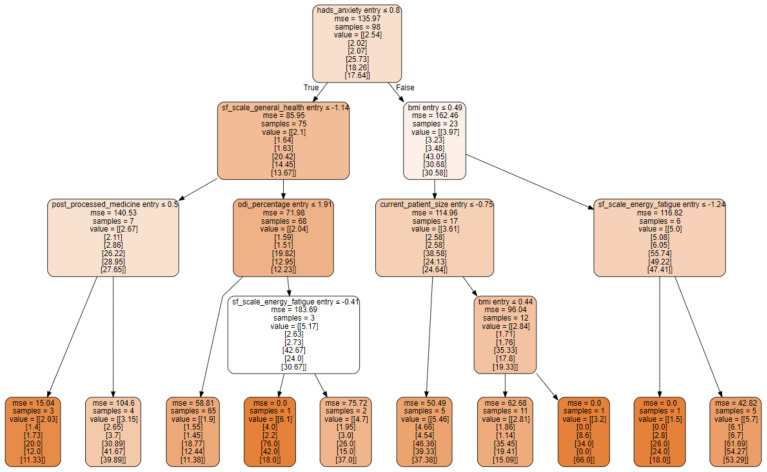
Visualisation of the way choices are made by the decision tree regressor algorithm used here.

Based on the importance of the individual components, the workflow of the algorithm used here can be depicted and the relevant decision paths are shown as a graphical overview (Figure 2). The order in which the algorithm processes and evaluates the individual parameters in order to produce a prediction can be identified here.

Interestingly, the establishment of a prediction does not start with the highest weighted influencing factor “BMI at admission” but with the value of the HADS anxiety score. Only at the next level are the BMI value and the SF 36 score for general health included.

## 4. Discussion

### 4.1. Interpretation of the Results in Relation to the Study Hypothesis

The interim results presented here are published as part of an ongoing study. The aim is to answer the hypothesis that there is a group of patients who would benefit from an early decision on the surgical treatment of a lumbar disc herniation. The establishment of an AI-based algorithm plays a decisive role in this context, in order to be able to ensure an optimal therapy decision by predicting the course of therapy at an early stage.

The algorithm presented here is able to provide a useful prediction of neuropathic pain (as a VAS value for leg pain) and complaints in everyday life (as an ODI score) caused by a lumbar disc herniation. The general applicability is still limited in its present form, but it provides a good enough quality to be used as an auxiliary tool. In particular, the combined prediction for several time points over the course of one year after the start of therapy allows comparative statements to be made for conservative or surgical measures.

### 4.2. Results in the Context of MCID

The results presented here have mean absolute errors for the VAS values ranging from 1.79 to 1.97. The minimum clinically important difference (MCID) is described in the literature with a wide range around 1.0 to 4.0. More recent work sees the MCID for the VAS around the value 1.65 to 2.0 on a scale of 0 to 10 [16,17,18]. Thus, the error values described here are still within the MCID and can be used as predictive values, especially if the predicted values deviate from the original values beyond the MCID.

For the ODI value, the MCID is described at about 9% [11,19,20]. With the mean absolute errors of 8.68 to 10.2 on a 100% scale using the algorithm presented here, the results of the ODI predictions are within a reasonable range. At least larger deviations between initial values and predictions beyond the value of the MCID can be used as an indicative value for therapy decisions.

### 4.3. Feature Importance Provides Clues to the Pathogenesis of Neuropathic Pain

It is frequently discussed which factors lead to a worse or better course of disease after lumbar disc herniation [3,4,6]. The machine learning algorithm established here is based on a decision tree regression that allows insight into the relevant factors. It is striking that the central starting point for a good prediction quality is the HADS anxiety score. This confirms the immense role of psychological factors on the pathogenesis of neuropathic pain due to lumbar disc herniation. The other important factors are the typical suspects. These include BMI and the general perception of one’s own health, followed by the extent of pre-existing diseases and medications as a measure of the actual pre-existing medical conditions. This confirms on the one hand known associations with the development of neuropathic pain, such as the influence of BMI [21,22], but on the other hand also reveals new aspects that need to be investigated in more detail in the further course of this study.

### 4.4. Role of Machine Learning as an Assisting Tool

There is currently growing interest in AI-based applications in the medical field. Above all, the prognosis of therapy outcomes represents an interesting option for dealing with questions that previously could not be addressed adequately [23,24,25].

The consideration of when surgical therapy of a lumbar disc herniation is advantageous and when conservative therapy can ensure an equivalent result has often been investigated, but the findings are difficult to interpret and often not applicable to everyday clinical practice [4,5,8,17]. Apart from relevant neurological deficits, the development of neuropathic pain due to radicular compression is the most important factor determining a patient’s permanent discomfort. The current literature often recommends a conservative treatment of these acute pain symptoms, since in a variety of studies conducted so far, comparable treatment results were observed for both treatment modalities after one year [1,2,6,26,27]. However, the study design of these investigations is often not comparable with clinical reality. Recently, several hints have also emerged that an early decision to invasive therapy measures can have advantages in the long-term course [4,5,8].

Within the framework of the ongoing study presented here, a machine learning algorithm is being established that is intended to enable an initial prediction of the further course of therapy. This enables the identification of patients at an early stage who may benefit from an early operative therapy. On the other hand, patients who cannot expect any benefit from surgical therapy can also be advised accordingly.

Comparable studies on the prediction of specific therapy outcomes after spinal surgery have now been published for several years with increasing practical prediction capabilities [9,28,29,30]. A gradual improvement of the predictions can be observed, which we attribute to improvements in the programming possibilities of algorithms as well as an improvement in data quality [31,32]. Further optimization of algorithms and perfection of data sets will, from our point of view, make it possible to establish them in a way that is suitable for everyday use in the future, and will also be possible in the context of the present study. We are convinced that the application of AI-based prediction of the therapy outcome, in treatment decision-making, will improve patient care and reduce the incidence of chronic neuropathic symptoms after lumbar disc herniation.

### 4.5. Limitations of the Study

The AI algorithm in the form presented here is an early stage of development. Relatively small numbers of patients were included. We were able to show earlier that it is possible to establish accurate prediction models even with low patient numbers [9]. However, a continuation of the study with a further increase in patient data sets will improve the accuracy of the predictions and thus make applicability more likely.

We see the main risks of bias or the occurrence of a wrong prediction in, on the one hand, an unclean data collection and, on the other hand, a local specificity of the data, as so far all data sets have been collected at a single spine center. For this reason, we are currently working on an expansion of the study to other spine centers in order to represent as broad a patient population as possible in our data collective. In addition, since the beginning of the study, we have tried to ensure that the risk of improper data collection, which must always be kept in mind, is eliminated through the use of repeated controlled data collection, blinded co-recording of the data sets and automated control processes generated through the used database.

As this is an ongoing study, the algorithm presented here should not be considered final, which can be seen as a limitation of this study. As new patient datasets are continuously included in this study and the algorithms are then continuously adapted, there is a dynamic change in the quality of prognosis, which cannot be regarded as definitive in the form published here. In general, the establishment of such a prediction based on an algorithm is necessarily subject to continuous change due to everlasting changes in the underlying patient population.

Thus far, no correlation of the predictions with image data is carried out. A corresponding adjustment is already planned, as every included patient has an MRI to confirm lumbar disc herniation. However, we do not expect that image data will cause a significant change in prediction quality. It has been shown previously that imaging and clinical complaints hardly correlate [33,34]. This can also be seen through our present results, which reflect the relevance of psychological factors and general comorbidities.

## 5. Conclusions

We present an ongoing study whose extensive datasets can be used to establish AI-based therapy predictions. A prediction quality in the range of the MCID is already achieved and can thus be used as an aid to advise patients with a lumbar disc herniation at an early stage. A reduction of neuropathic pain through early establishment of individually adapted therapies is thus possible.

## Figures and Tables

**Table 1:** Continuous patient data (demographic and clinical scores) on admission.

	*n* = 123			
	Mean	Std	Min	Max
**Age**	53.2	13.2	27	83
**Days from onset of symptoms to 1st treatment**	29.3	27.4	1.0	84.0
**BMI**	27.6	6.1	17.9	54.7
**Patient standing height**	173.5	9.8	152.0	196.0
**Pack years (smoking)**	7.3	12.7	0.0	47.0
**Lasègue**	42.7	15.8	5.0	60.0
**SF 36 physical functioning**	24.0	21.8	0.0	95.0
**SF 36 limit. physical health**	15.4	29.6	0.0	100.0
**SF 36 limit. emotional problems**	50.4	46.4	0.0	100.0
**SF 36 energy fatigue**	39.1	18.1	5.0	90.0
**SF 36 emotional well being**	60.6	19.3	20.0	100.0
**SF 36 social functioning**	54.4	26.7	0.0	100.0
**SF 36 pain**	21.9	23.7	0.0	100.0
**SF 36 general health**	57.8	19.1	10.0	100.0
**ODI percentage**	57.0	19.5	11.1	100.0
**HADS anxiety**	6.6	3.9	0.0	15.0
**HADS depression**	6.5	3.5	0.0	15.0
**Back pain (VAS)**	5.7	3.0	0.0	10.0
**Leg pain (VAS)**	5.5	3.4	1.1	10.0

**Table 2:** Categorical patient data on admission.

Gender	Male	Female	
***n* = 123**	69	54	
**Treatment option**	**Operation**	**Conservative**	**Cross-over**
***n* = 123**	52	37	34
**Neurological deficit on admission**	**Motor weakness**	**Sensory deficit**	
***n* = 123**	43 out of 123	77 out of 123	

**Table 3:** Variables identified and used for final training.

Categorical Variables	Continuous Variables on Admission	Target Variables
motor weakness	Age	ODI after 6 weeks
Treatment option	Days from onset of symptoms to 1st treatment	ODI after 6 months
	SF 36 limitations physical health	ODI after 1 year
	SF 36 limitations emotional problems	Leg pain after 6 weeks
	SF 36 pain	Leg pain after 6 months
	Patient standing height	Leg pain after 1 year
	SF 36 emotional well being	
	SF 36 social functioning	
	SF 36 physical functioning	
	SF 36 general health	
	Back pain	
	Leg pain	
	BMI	
	HADS anxiety	
	ODI	
	Days from admission to cross over	
	Pre-existing conditions and medication types	

**Table 4:** Performance results (combined mean absolute error, MAE for all 6 target values) of various other machine learning algorithms obtained in 10-fold cross validation.

Machine Learning Algorithm	MAE	SD
Linear Regression	9.19	0.37
Elastic Net	9.57	0.89
Nearest Neighbour	8.13	0.37
Random Forest	7.51	0.02
Neuronal Net	8.11	0.54

**Table 5:** Combined mean absolute error (MAE) and mean and standard deviation of the 10-fold cross-validation for the prediction of all target values using our best performing decision tree regressor model.

Fold	1	2	3	4	5	6	7	8	9	10	Mean	SD
MAE	7.33	8.10	6.23	6.34	7.71	7.61	8.68	5.75	6.76	5.70	7.02	0.97

**Table 6:** Detailed mean absolute error of the performed 10-fold cross-validation for the prediction of each target value using our decision tree regressor model.

Fold	1	2	3	4	5	6	7	8	9	10	Mean	SD
VAS leg pain 6 weeks	2.16	1.68	1.67	1.21	1.55	1.75	2.10	2.23	2.01	1.56	1.79	0.31
VAS leg pain 6 months	2.25	2.13	1.82	1.66	1.11	2.04	1.87	1.43	2.45	1.37	1.81	0.40
VAS leg pain 1 year	2.58	1.91	2.07	2.25	1.58	1.85	2.25	1.52	2.06	1.60	1.97	0.33
ODI value 6 weeks	9.12	9.59	9.46	9.31	11.74	10.39	11.68	7.22	5.87	10.71	9.51	1.75
ODI value 6 months	11.96	10.22	8.84	10.35	9.20	9.26	9.72	13.79	8.83	7.99	10.02	1.62
ODI value 1 year	9.55	8.40	7.78	11.61	8.97	8.57	7.16	9.77	7.48	7.52	8.68	1.29

## Data Availability

The original data as well as the algorithms used in the present study can be reviewed upon reasoned request via the corresponding author.
